# Differential Anti-Fibrotic and Remodeling Responses of Human Dermal Fibroblasts to *Artemisia* sp., Artemisinin, and Its Derivatives

**DOI:** 10.3390/molecules29092107

**Published:** 2024-05-02

**Authors:** Pamela Weathers, Melissa Towler, Bushra Hafeez Kiani, David Dolivo, Tanja Dominko

**Affiliations:** Department of Biology and Biotechnology, Worcester Polytechnic Institute, Worcester, MA 01609, USA; eeyore@wpi.edu (M.T.); bushra.hafeez@iiu.edu.pk (B.H.K.); david.dolivo@gmail.com (D.D.); tdominko@wpi.edu (T.D.)

**Keywords:** artemisinin, artesunate, dihydroartemisinin, artemether, *Artemisia annua*, *Artemisia afra*, tea infusion, α-SMA

## Abstract

Fibrosis is a ubiquitous pathology, and prior studies have indicated that various artemisinin (ART) derivatives (including artesunate (AS), artemether (AM), and dihydroartemisinin (DHA)) can reduce fibrosis in vitro and in vivo. The medicinal plant *Artemisia annua* L. is the natural source of ART and is widely used, especially in underdeveloped countries, to treat a variety of diseases including malaria. *A. afra* contains no ART but is also antimalarial. Using human dermal fibroblasts (CRL-2097), we compared the effects of *A. annua* and *A. afra* tea infusions, ART, AS, AM, DHA, and a liver metabolite of ART, deoxyART (dART), on fibroblast viability and expression of key fibrotic marker genes after 1 and 4 days of treatment. AS, DHA, and *Artemisia* teas reduced fibroblast viability 4 d post-treatment in up to 80% of their respective controls. After 4 d of treatment, AS DHA and *Artemisia* teas downregulated *ACTA2* up to 10 fold while ART had no significant effect, and AM increased viability by 10%. *MMP1* and *MMP3* were upregulated by AS, 17.5 and 32.6 fold, respectively, and by DHA, 8 and 51.8 fold, respectively. ART had no effect, but *A. annua* and *A. afra* teas increased *MMP3* 5 and 16-fold, respectively. Although *A. afra* tea increased *COL3A1* 5 fold, *MMP1* decreased >7 fold with no change in either transcript by *A. annua* tea. Although *A. annua* contains ART, it had a significantly greater anti-fibrotic effect than ART alone but was less effective than *A. afra*. Immunofluorescent staining for smooth-muscle α-actin (α-SMA) correlated well with the transcriptional responses of drug-treated fibroblasts. Together, proliferation, qPCR, and immunofluorescence results show that treatment with ART, AS, DHA, and the two *Artemisia* teas yield differing responses, including those related to fibrosis, in human dermal fibroblasts, with evidence also of remodeling of fibrotic ECM.

## 1. Introduction

Fibrosis is a pathological healing process where the response to tissue injury occurs via non-regenerative mechanisms and leads to scar formation [1]. Fibrotic healing can affect a variety of organs, including the skin, liver, kidney, lungs, and heart. Instead of replacing injured tissue with functional native cellular components and an appropriate microstructure, this chronic condition is characterized by transdifferentiation of resident connective tissue fibroblasts or other progenitor cells into highly synthetic and contractile α-SMA^+^ myofibroblasts [2,3], which produce excessive amounts of acellular, primarily collagen I-based, extracellular matrix (ECM) that lacks functional properties of the uninjured tissue. As fibrosis develops in myriad organs in response to many different types of tissue stress and damage, common estimates suggest that nearly half of all deaths in the developed world are consequent to the development of tissue fibrosis [4], while fibrotic conditions that do not result in mortality may still confer substantial morbidity. Although our understanding of the basic science of fibrosis has progressed significantly over the past several decades, the development of clinically successful therapeutics has lagged greatly behind the elucidation of key mechanisms underlying the development, progression, and maintenance of tissue fibrosis (reviewed by Henderson et al., 2020 [5]), underlying a dire need for the discovery of novel anti-fibrotic therapeutics.

**Figure 1:**
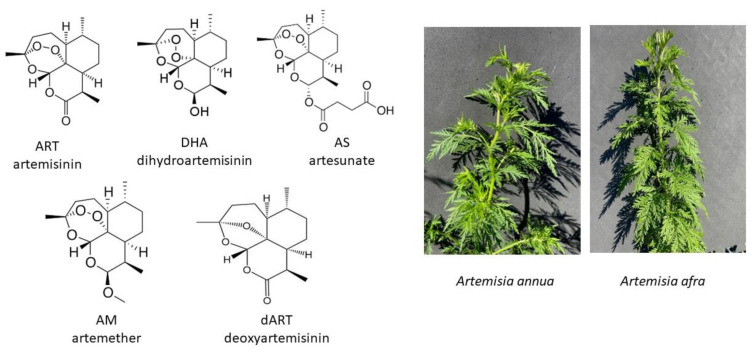
(**Left**) artemisinin (ART), artemisinin derivatives (DHA, AS, AM), and an artemisinin liver metabolite, deoxyartemisinin (dART); (**right**) *Artemisia annua* and *afra*.

Artemisinin (ART), produced in the plant *Artemisia annua* L. (Figure 1), and ART derivatives (artesunate, AS; dihydroartemisinin, DHA; and artemether, AM), are sesquiterpene lactone antimalarials with demonstrated antiparasitic [6,7], antibacterial [6,8,9,10], antiviral [11,12,13,14], anticancer [15], and anti-fibrotic effects [16,17,18]. Previously, AS treatment was shown to ameliorate the fibrotic response in human dermal fibroblasts [17], which is consistent with the broader collection of the literature describing the anti-fibrotic effects of ART compounds in the fibrotic progenitor cells of other organs (reviewed by Dolivo et al., 2021 [16] and Henderson et al., 2020 [5]). Despite these encouraging initial findings, there remains a lack of understanding regarding the mechanisms by which artemisinic drugs exert their anti-fibrotic effects. Also lacking is an understanding of the similarities and differences among the anti-fibrotic responses to various ART compounds, complicating the use of these compounds for further study and clinical development to combat fibrotic clinical indications. When compared, *A. annua* is equally or more efficacious in vitro than ART against many of the same diseases [19,20], e.g., tuberculosis (TB) [8,9] and COVID-19 [13,21]. ART from *A. annua* also distributes efficiently to many tissues and organs, including the lungs, liver, muscles, brain, and heart [22]. A side benefit of using ART or *A. annua* is their antinociceptive activity [23,24,25,26,27]. There also are no significant adverse effects with long-term use of *A. annua* [23,24,25,28]. Though *A. annua* is globally used as a medicinal plant against many diseases including malaria, and despite accumulating evidence that ART chemical derivatives may be effective in counteracting fibrotic tissue responses, there is a dearth of studies examining the effects of *A. annua*, in particular on fibrosis.

Here we compared the effects of ART, AS, DHA, AM, and *A. annua* and *A. afra* hot-water extracts (tea infusions) on the viability and gene expression of dermal fibroblasts. Tea infusions remain a common mode of traditional use among global populations, especially in low- and middle-income countries [29], and evidence exists that additional phytochemicals present in *Artemisia* tissue may further contribute to their therapeutic effects in other diseases [19,20]. Thus, a study of the effects of ART delivered as a tea infusion may shed light on the potential beneficial effects of a therapeutic modality already widely in use. Comparisons among ART derivatives, as well as comparisons to hot-water extracts, enable a better understanding of which drugs are more optimal for the treatment of fibrosis, whether through application directly or after further derivatization or formulation development. Information regarding these comparisons may also aid in the interpretation of the existing literature that seeks to investigate the effects of various ART compounds on fibrosis, as these reports have sought to use the myriad of different ART compounds without assessing the comparative efficacy against other compounds, nor giving much explanation as to their choice of one compound over the others.

## 2. Results

### 2.1. Differential Effects of Artemisia Teas vs. ART Drugs on Human Dermal Fibroblast Viability

**Figure 2:**
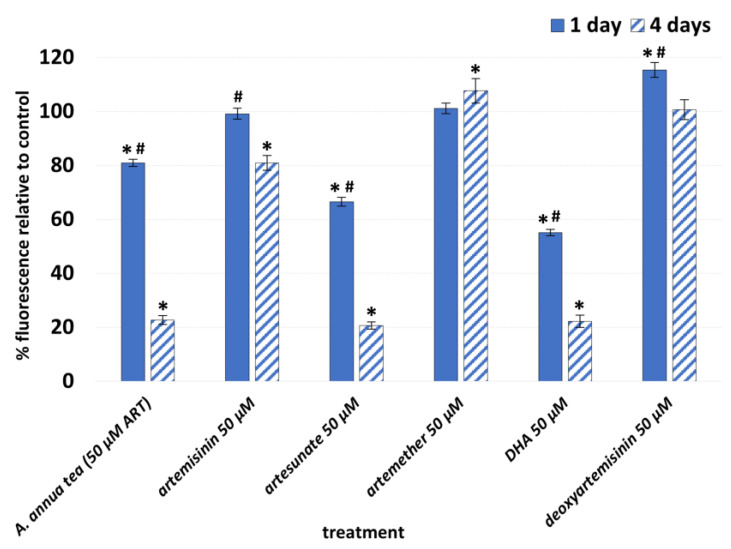
Relative viability responses of human dermal fibroblasts via resazurin assay at 1 and 4 days after exposure to artemisinin, each of the artemisinic derivatives, artemisinin’s liver metabolite deoxyartemisinin, and hot-water extracts of *A. annua*, all normalized to 50 µM of the relevant artemisinic compound. Control = 100%; N ≥ 12; * = *p* ≤ 0.05 for samples to solvent control; # = *p* ≤ 0.05 for 1 day compared to 4 days using Student’s *t*-test.

Preliminary data from viability assays suggested that *A. annua* tea, ART, and AS did not similarly affect the viability of human dermal fibroblasts. That led to further exploration of how the other ART derivatives, AS, AM, and DHA, and ART’s liver metabolite dART, affected fibroblast viability. After 1 d of treatment, fibroblast viability significantly decreased by about 20, 35, and 45% of their solvent controls after treatment with *A. annua* tea, AS, or DHA, respectively (Figure 2). In contrast, after 1 d exposure to ART or AM, there was no significant change in fibroblast viability. Treatment with dART, a liver metabolite of ART, yielded a ~15% increase in viability. At 4 d post-treatment, cell viability decreased in treatments with ART, AS, DHA, and *A. annua* tea by about 20, 80, 78, and 77% of their controls, respectively (Figure 2). After 4 d treatment with AM, cell viability increased by nearly 10%, but dART showed no change compared to its control (Figure 2). Together these results showed that, while ART is a major constituent of *A. annua* tea, it had either no or only a slight effect on fibroblast viability as a pure molecule at the same molar concentration as in the tea. In contrast, *A. annua* tea, AS, and DHA all significantly inhibited fibroblast viability. Further experiments focused on the effects on the expression of some fibrosis-related genes by *A. annua* tea, ART (to compare with the ART in *A. annua* tea), AS, and DHA.

### 2.2. Differential Expression of Fibrotic Genes in ART-Drug-Treated Fibroblasts

The preliminary transcriptional responses of eight genes (*ACTA2*, *COL1A1*, *COL3A1*, *TGFB1*, *TGFB2*, *TGFBR1*, *MMP1*, and *MMP3*) related to ECM remodeling and transition to fibrosis were measured in fibroblasts after 4 d of treatment with ART, AS, or a hot-water extract of *A. annua*. Transcriptional results showed that cells treated with ART or AS responded differently, with AS having a more robust effect on transcription than ART. ART and *A. annua* tea treatments also yielded an unexpectedly dissimilar response, given that we had hypothesized that the effects of *A. annua* tea on fibroblasts were due at least in part to the effects of ART.

**Figure 3:**
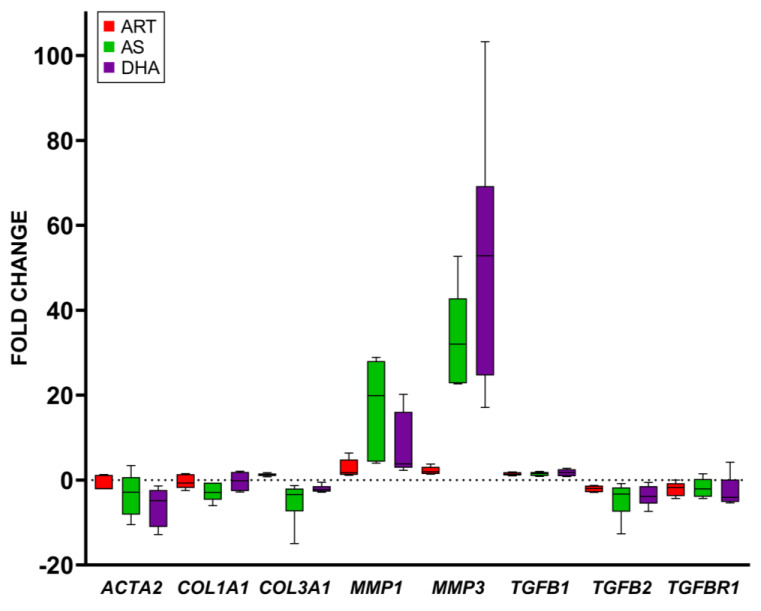
Fold change vs. solvent control of gene expression in fibroblasts after 4 d of treatment with 50 µM ART, AS, or DHA; *GAPDH* was used as the internal control; N ≥ 5.

Because they did not inhibit fibroblast viability (Figure 2), AM and dART were eliminated from further study, and instead, the focus was on transcriptional analyses of the effects of ART, AS, and DHA (Figure 3). After fibroblasts were treated for 4 d with 50 µM ART, AS, and DHA, ART increased *MMP1* about 2.8 fold but otherwise lacked effects on the expressions of other markers. AS decreased *ACTA2* expression by about 3.4 fold, *COL1A1* 2.9 fold, *COL3A1* 5 fold, *TGFB2* 4.6 fold, and *TGFBR1* 2.5 fold. AS increased *MMP1* and *MMP3* 17.5 and 32.6 fold, respectively. DHA decreased *ACTA2* expression 6.2 fold, *TGFB2* 3.7 fold, and *TGFBR1* 4 fold, while increasing *MMP1* and *MMP3* 8 and 51.8 fold, respectively. Other investigated marker genes were unaffected.

### 2.3. A. afra vs. A. annua

**Figure 4:**
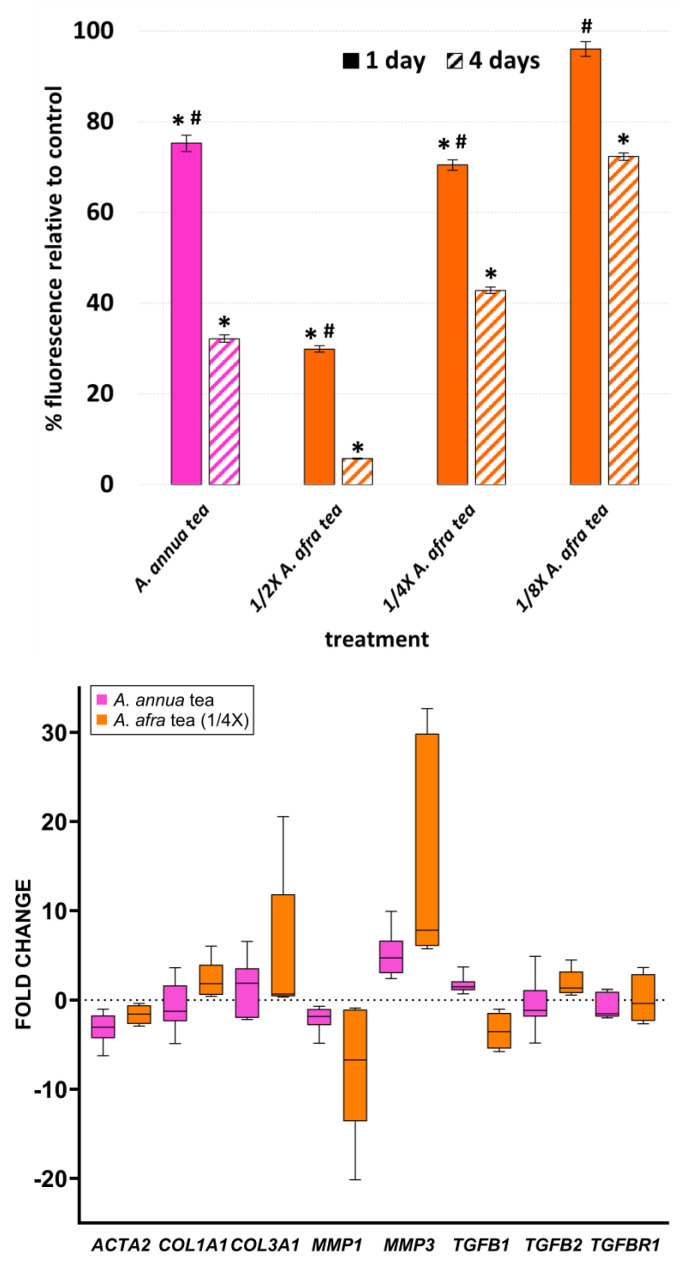
Viability and transcript expression of fibroblasts after treatment with *A. annua* or *A. afra* infusions. (**Top**) resazurin assay of fibroblast viability after 1 and 4 d treatment with *A. annua* and *A. afra* infusions. (**Bottom**) Fold change vs. solvent control of gene expression in fibroblasts after 4 d treatment with infusions of *A. annua* (at 50 µM ART content) and *A. afra* diluted to ¼ of the dry mass of the *A. annua* tea; *GAPDH* was used as the internal control; N = 12 for resazurin assay (* = *p* ≤ 0.05 vs. solvent control; and # = *p* ≤ 0.05, 1 d vs. 4 d); and N ≥ 4 for gene expression.

*A. afra* lacks ART but is closely related to *A. annua*, and it has shown similar therapeutic efficacy against various diseases. Therefore, to better query whether other phytochemicals may be affecting fibroblast viability and gene expression, we compared infusions of *A. afra* to those of *A. annua* (Figure 4). Surprisingly, on a dry-mass basis, *A. afra* was about four times more potent than *A. annua* at inhibiting fibroblast viability (Figure 4) and, at a concentration equal to that of the *A. annua* infusion, completely inhibited fibroblast viability. Transcriptional responses were also dramatically different, with quarter strength *A. afra* tea increasing *COL3A1* transcripts by about 5 fold compared to untreated cells, whereas *A. annua* tea did not affect *COL3A1* expression. *A. afra* also increased *MMP3* expression nearly 16 fold (versus 5 fold for *A. annua*) but decreased *MMP1* expression 7.2 fold and *TGFB1* 3.5 fold. There was no effect of *A. annua* on *MMP1*.

### 2.4. Differential Expression of α-SMA Protein after 1 and 4 Days of ART, ART Drugs, and Artemisia Treatments in Fibroblasts

**Figure 5:**
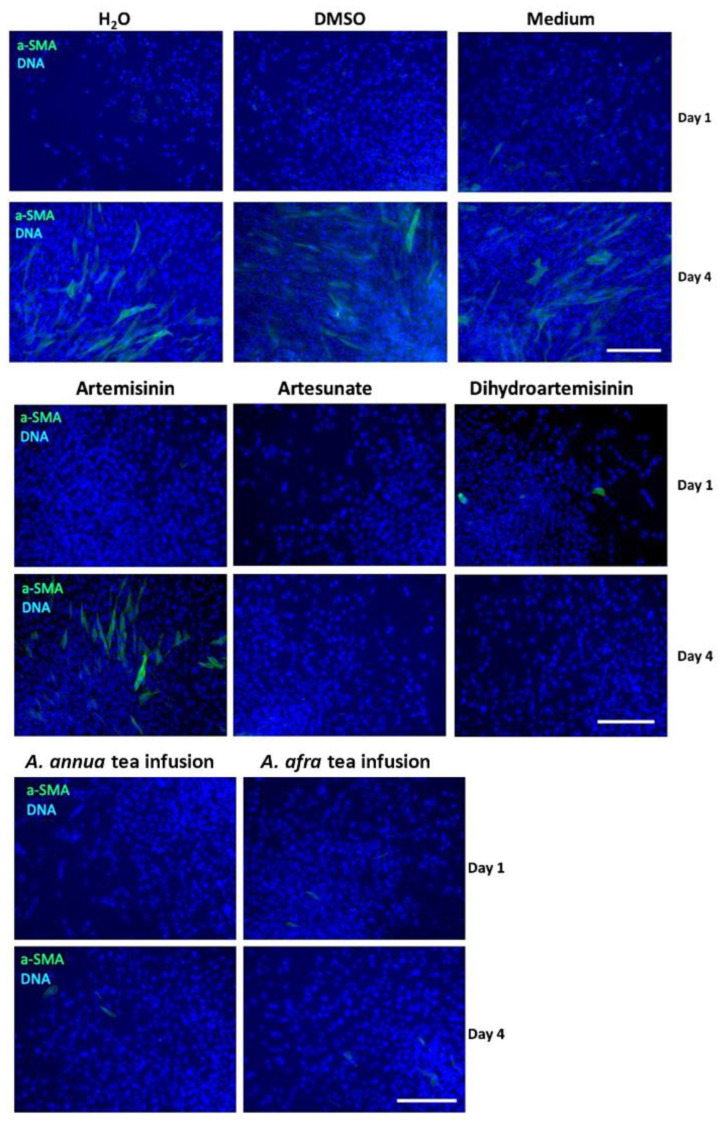
Representative views of fibroblasts after 1 and 4 d treatment with ART, AS, DHA, and *A. annua* and *A. afra* (1/4X) tea infusions (*N* = 3). Cells were stained for smooth-muscle α-actin (α-SMA, green) and nuclei were counterstained with Hoechst (blue). Scale bar = 100 μm for all images.

To determine if transcriptional changes were also accompanied by changes in protein levels, fibroblasts treated for 1 and 4 d with drugs were fixed and stained for α-SMA using immunofluorescence. DMSO (control solvent for all ART pure drugs), water (control solvent for *Artemisia* teas), and media with no solvent or drug showed similar staining patterns of α-SMA (Figure 5). After 4 d, α-SMA staining correlated with the 4 d qPCR data in Figure 3 and Figure 4; AS, DHA, and the two *Artemisia* teas considerably decreased α-SMA compared to their respective solvent controls (Figure 5). In contrast, ART showed a substantial increase in α-SMA staining compared to its untreated DMSO control. Together, our qPCR and immunofluorescence results correlate, demonstrating that ART, AS, DHA, and the two *Artemisia* teas have different anti-fibrotic responses in human dermal fibroblasts.

### 2.5. A. annua, ART, and ART Drugs Show Bioconversion in Culture Medium after 4 d Incubation

**Figure 6:**
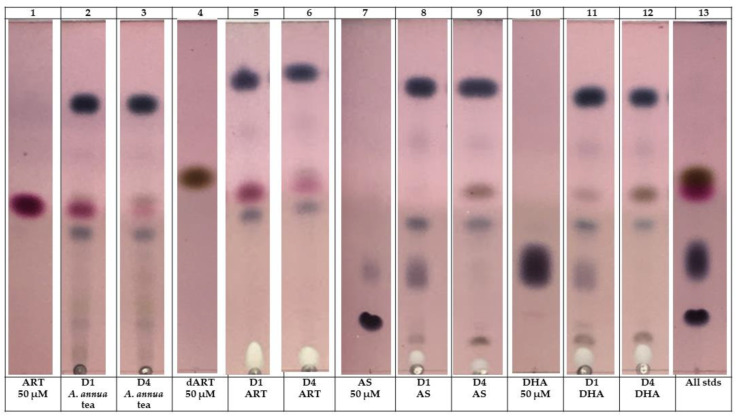
Thin-layer chromatography of media after 1 and 4 days *A. annua* tea and ART drug treatments of fibroblasts. Media were extracted with dichloromethane, spotted on Si-gel 60 F_254_ plates, and run in a mobile phase of 5:11:4 DCM:hexane:acetone plus 0.5% *v*/*v* acetic acid. Plates were stained with *p*-anisaldehyde reagent that stains all ART drugs dark blue–purple, except for ART (dark pink) and dART (brown). Each D1 or D4 spot represents 1 mL of extracted media, and the ART, dART, AS, and DHA standards are the equivalent of 1 mL of a 50 µM solution. Image is a composite of representative media extracts of three replicates.

Microscopic analysis of fibroblasts during treatment suggested that differences among the effects of ART drugs may be mediated by the presence of particular artemisinic metabolic products in culture, especially in those treated with DHA and AS. We consequently extracted media from cells incubated for 1 and 4 d in media containing *A. annua* tea (diluted to 50 µM ART), and ART, AS, or DHA at 50 µM as for previous experiments, and then separated the extracts via thin-layer chromatography (TLC) to visualize the amount of each ART drug remaining after 1 and 4 d treatment (Figure 6). When treated with *A. annua* tea, ART nearly disappeared from the media after 4 d of drug treatment and dART became more apparent, but not in amounts sufficient to account entirely for the ART disappearance if converted to dART; also note that dART is already present in *A. annua* tea (Table 1). A similar disappearance of ART from ART-treated cells occurred, accompanied by an increase in dART, by day 4. AS and DHA were nearly gone from the media after day 1 and were undetectable by day 4 (Figure 6), suggesting that any effects from these compounds were likely driven by responses initiated early in the treatment period.

Because fibroblast culture medium has a diversity of components, we wondered if the medium itself contributed to the degradation of the drugs. This was confirmed after GC-MS analysis of the culture media incubated with the same concentrations of drugs for 4 days, but without any cells (Table 1). Of the ART in the *A. annua* tea extract, ~9% remained on day 4, but >99% of ART in the ART-containing medium disappeared. Neither AS nor DHA was detectable after 4 d of incubation in cell-free media (Table 1). The CYP P450 metabolite of ART, dART, was observed in AS and DHA samples after 4 d of drug treatment (Figure 6). Surprisingly, fibroblast-free media also had dART after 4 d incubation in drugs (Table 1) suggesting P450 activity in the culture media, which does contain some serum. Taken together, however, the media analyses indicate that the dramatic differences observed in viability, gene expression, and immunochemistry among cell cultures exposed to different ART drugs and *Artemisia* tea treatments are likely regulated by different metabolites of these species in culture, at least some of which occurs even in the absence of cells. This suggests that more frequent drug dosing and monitoring of media metabolites is essential in order to better understand the in vitro efficacy of ART drugs.

## 3. Discussion

Fibrosis is a pathological process of staggering consequence. The development of effective treatments for tissue fibrosis has been hampered by the clinical failure of agents designed toward obvious fibrotic targets, particularly TGF-β family ligands and receptors, largely due to functional redundancy and positive feedback loops characteristic of the fibrotic response [30]. In addition, therapeutic strategies that are shown to blunt the development of tissue fibrosis in pre-clinical models may translate poorly to use in humans, since human patients tend to be diagnosed with fibrotic indications at late stages of disease development, at which time fibrosis has advanced significantly and resulted in tissue damage, necessitating reversal rather than prevention of the fibrotic response in order to yield any functional benefit [31]. Therefore, drugs that can slow the development and progression of fibrosis in humans are of key interest. Accumulating evidence yielded by preclinical in vitro and in vivo models has pointed towards ARTs as potentially efficacious compounds to combat tissue fibrosis, and decades of use of ARTs for malaria have demonstrated an impressive record of safety and have afforded a broad understanding of their pharmacology. Therefore, one major aim of the current work is to better understand the effects of these compounds on processes critical to tissue fibrosis.

Against malaria, the effects of ARTs are consistent, requiring the endoperoxide bridge, acting via several mechanisms. As a prodrug, ART is activated by heme after parasite digestion of hemoglobin, resulting in the generation of reactive oxygen species (ROS) [32] that damage the parasite but not the human cells (see review by Yang et al. [33]). In another case, the mitochondria of the parasite activate ART, leading to mitochondrial damage to the parasite but not to human cells [33]. Other possible mechanisms include the inhibition of PfATP6, analogous to mammalian SERCA, and the potential covalent binding of heme-activated ART to a large number of other identified proteins [33,34,35]. AS, DHA, AM, and ART all contain the endoperoxide bridge responsible for the heme activation of ART (Figure 1). However, in dermal fibroblasts, this study showed that neither ART nor AM seemed to have anti-fibrotic or potential ECM-remodeling activity, while AS, DHA, and *Artemisia* extracts did, suggesting that presence of the endoperoxide bridge in the ART molecule is not sufficient to yield the observed activity on fibroblasts. Furthermore, ART-containing *A. annua* has in vitro anti-fibrotic activity that differs from pure ART, suggesting that the plant tissue contains other molecules with the ability to modulate fibrotic responses, potentially justifying consideration of *Artemisia* plant tissue as an alternative therapeutic modality for fibrosis compared to the pure drug.

The degradation of AS to DHA and then DHA to dART reflects the instability of AS in aqueous media [36]. AS is a form of ART acylated with succinate in order to make the drug more bioavailable, but it is also readily hydrolyzed to DHA, which is the bioactive metabolite against malaria (see review by Morris et al. [37]). Although the observed degradation of ART drugs over 4 d may indicate a need for additional dosing in future experiments, the data showed that there were still measurable phenotypic responses of cultured fibroblasts to the drugs. Nevertheless, the observed degradation should be considered in any analysis of subsequent results, as well as a lens through which to interpret other data generated from the use of these drugs in cell culture. For example, while a variety of ART drugs, including ART, DHA, AM, AS, and SM934, have demonstrated anti-fibrotic efficacy when applied to various disease models in vivo, the vast majority of in vitro data in the cell types relevant to fibrosis have been generated using AS or DHA (reviewed by Dolivo et al. [16]). Notably, this is consistent not only with the data in this manuscript which, for example, demonstrated anti-proliferative effects towards fibroblasts from AS and DHA that were notably lacking in ART and AM, but also with the known susceptibility of AS to spontaneous (non-enzymatic) decomposition in an aqueous solution [38] or (enzymatic) hydrolysis of the ester group in the presence of esterases (e.g., introduced by animal serum supplemented to the culture medium). This raises the intriguing possibility that the active anti-fibrotic agent common to most (or even all) of these previous studies is DHA, regardless of the specific agent delivered, and that myriad reports of the anti-fibrotic effects of ART drugs both in vivo and in vitro are actually describing the delivery of pro-drugs that only ameliorate fibrosis when they result in the administration of meaningful quantities of DHA (i.e., (1) via aqueous decomposition or enzymatic conversion to DHA in vitro, depending on the cell and media type, (2) via conversion to DHA in vivo, or (3) due to direct administration of DHA as an active component). Further metabolism of AS, ART, and DHA to dART in cell-free media may be from the trace cytochrome P450 activity of serum in fibroblast culture media [39]. Further study will seek to understand the relationship between conversion among these chemical species and their therapeutic effects, as well as to use this knowledge to select or design the most optimal ART drugs for treating fibrosis.

Here it was demonstrated that administration of AS, DHA, or *Artemisia* teas limited cellular proliferation and resulted in the decreased expression of myofibroblast marker genes and α-SMA protein. This suggested that these compounds may limit fibroblast proliferation and formation of myofibroblasts in the wound-healing cascade, processes critical to the development of tissue fibrosis. Additionally, the observed increase in *MMP3* transcripts by AS, DHA, and *A. afra* and increases in *COL3A1* by *A. afra* also indicate these drugs have the potential to affect the remodeling of the ECM. Given the critical role of the ECM in directing cellular and tissue processes dictating regenerative and fibrotic healing [40], and understanding that fibroblasts are major cellular agents regulating ECM deposition and remodeling (see Henderson et al., 2020 [5]), further research will seek to uncover the effects of ARTs on the deposition of specific ECM proteins and ECM-remodeling enzymes in order to better understand the processes underlying the demonstrated anti-fibrotic properties of ARTs in vivo.

Extensive research has identified ART derivatives as effective therapeutics for blunting the development of tissue fibrosis in both in vitro and pre-clinical animal models of disease (reviewed in [16]). However, practically none of this literature has investigated the therapeutic effects of ART delivered as *A. annua*, nor the additional benefit of other phytochemicals introduced upon the ingestion of plant material. Previously, ART was shown to be significantly more bioavailable when delivered per os via the plant vs. as a pure drug and distributed to multiple organs including the heart, lungs, liver, and brain [22]. This greater ART bioavailability from the plant vs. the pure drug is enhanced by the plant’s essential oils [41,42], greater intestinal transport [43], and inhibition of hepatic CYPs 3A4 and 2B6 metabolism of ART [22,44], resulting in greater levels of serum ART [22,45,46].

Limitations of this study include investigation of the expression of a small number of genes and proteins, as well as analysis of only one isolate of human fibroblasts, though our data are consistent with that of other demonstrations of similar effects in other isolates of fibroblasts [17,47,48], as well as with numerous reports of the anti-fibrotic activity of AS and DHA in other myofibroblast progenitor cells that are known to contribute to fibrosis. Future work will seek to characterize with greater resolution the effects of ART compounds and *Artemisia* extracts on pro-fibrotic phenotypes in fibroblasts, as well as assess and compare the anti-fibrotic effects of these treatments utilizing in vivo models of skin fibrosis, which will shed light on the effects of these compounds on other cells relevant to wound healing (e.g., macrophages, keratinocytes, endothelial cells) and provide evidence for or against their use as therapeutic compounds for skin fibrosis in humans.

## 4. Materials and Methods

### 4.1. Biological Materials

CRL-2097 human dermal fibroblasts were acquired from ATCC and maintained in culture in DMEM/F-12 with GlutaMAX supplement (Thermo Fisher Scientific, Waltham, MA, USA, 10565018) and 10% Fetal Clone III (Cytiva, Marlboro, MA, USA, SH30109.03) and incubated at 37 °C and 5% CO_2_ in high humidity. Dried leaves of *Artemisia annua* L. cv. SAM (voucher MASS 317314; batch B#1.Sh6.01.15.20) and *Artemisia afra* cv. MAL Jacq. ex Willd. (voucher FTG181107; batch B#1RbA.10.12.20) were acquired from Atelier Temenos LLC in Homestead, FL, USA. Hot-water extracts (tea) at 10 g leaf dry weight per L were prepared according to Kane et al. [44]. The ART content in the tea was 581.69 µM, as analyzed by the method described in Martini et al. [9], and was diluted to 50 µM for the experiments. For the *A. afra* experiments, hot-water extracts were prepared in a similar manner.

### 4.2. Drug Treatments

Drug treatments were performed as follows. *A. annua* tea was diluted in a growth media to 50 µM ART; *A. afra* tea was diluted with water to 1/2, 1/4, or 1/8 strength of the *A. annua* tea on a dry-weight basis as needed; artemisinin (ART; Cayman Chemical, Ann Arbor, MI, USA, 11816), artesunate (AS; Cayman Chemical 11817), artemether (AM; gift from Prof. J. Plaizier-Vercammen (Brussels, Belgium)), dihydroartemisinin (DHA; Cayman Chemical 19846), and deoxyartemisinin (dART; Toronto Research Chemicals, Toronto, Ontario, Canada, D232150) were dissolved in DMSO as 1000× stock solutions, filter sterilized, and added to a final concentration of 50 μM. DMSO and water were the solvent controls for the artemisinic compounds and teas, respectively. Fibroblasts (passages 10–14) were seeded in 6-well plates in 2 mL medium at a density of 6250 cells/cm^2^. After overnight attachment, media were aspirated and replaced with test drugs in growth media at the indicated concentrations and incubated at 37 °C and 5% CO_2_ in high humidity. After 4 d of drug treatment, cells were harvested, washed with 1× Dulbecco’s phosphate-buffered saline (DPBS; Thermo Fisher Scientific 14190136), snap frozen in liquid nitrogen, and stored at −80 °C until the time of RNA extraction for analysis.

### 4.3. Resazurin Assay

Fibroblasts (passages 9–11) were seeded in 96-well plates at a density of 2000 cells/well (~6060 cells/cm^2^) and, after overnight attachment, cells were treated with drugs as already described. After 1 or 4 d of treatment, media were aspirated and replaced with media containing resazurin (Thermo Fisher Scientific B21187.03) at a final concentration of 25 µg/mL (stock solution 0.15 mg/mL in DPBS). Cells were incubated for 2 h at 37 °C as above, and then fluorescence was read at ex/em = 544 nm/590 nm on a Perkin Elmer Victor3 1420 plate reader. Fluorescence readings were normalized to the appropriate solvent controls.

### 4.4. RNA Isolation and Quantitative Real-Time Polymerase Chain Reaction (qRT-PCR)

RNA from cells after 4 d of drug treatment was isolated using the NucleoSpin RNA/protein mini kit (Macherey-Nagel, Allentown, PA, USA, 740933.50) according to the manufacturer’s instructions. The concentration of isolated RNA was measured using a Nanodrop 2000c Spectrophotometer (Thermo Scientific). Complementary DNA (cDNA) was synthesized from RNA using a QuantiTect reverse transcription kit (Qiagen, Germantown, MD, USA, 205313) following the manufacturer’s instructions. A SYBR-based PowerTrack master mix (Thermo Fisher Scientific A46012) was used to perform RT-qPCR on a QuantStudio 6 Pro Real-Time PCR system (Thermo Fisher Scientific) in 10 μL reactions. The thermal protocol was as follows: 95 °C for 2 min, then 40 cycles of 95 °C for 15 s and 60 °C for 60 s. PCR primer sequences are listed in Table 2 and were the same as used in Larson et al., 2019 [17]. Fold change in gene expression was determined using the ΔΔCT method [49] with GAPDH as the housekeeping gene.

### 4.5. Immunocytochemistry

Fibroblasts were seeded in 48-well plates at 6060 cells/cm^2^ and, after overnight attachment, cells were treated with drugs as described above. After 1 and 4 d, cells were fixed with paraformaldehyde and stained for immunofluorescence using primary mouse anti-α-SMA (Santa Cruz Biotechnology, Dallas, TX, USA, sc-32251) and secondary goat anti-mouse IgG AlexaFluor488-conjugated antibodies (Thermo Fisher Scientific A-11001), and DNA was counterstained with Hoechst 33342 as previously described [50].

### 4.6. Analysis of Artemisinic Metabolites in Media

Media were extracted 1:1 with dichloromethane overnight, after which the solvent was separated and dried. Extracts (equivalent to 1 mL media) were resuspended in dichloromethane, spotted on Si-gel 60 F254 plates (EMD Millipore, Burlington, MA, USA, cat #1.05735.0001), and run in a mobile phase of 5:11:4 dichloromethane–hexane–acetone plus 0.5% *v*/*v* acetic acid. Plates were stained with *p*-anisaldehyde reagent (75 mL ethanol, 2.5 mL sulfuric acid, 1 mL acetic acid, and 2 mL *p*-anisaldehyde (Sigma Aldrich, St. Louis, MO, USA, A88107-100G)) and heated for 10 min at 105 °C. This reagent stains all ART drugs dark blue–purple, except for ART (dark pink) and dART (brown). ART, dART, AS, and DHA were quantified in fibroblast media after two-phase extraction with dichloromethane using gas chromatography–mass spectrometry as previously detailed in Kane et al. [44] on an Agilent GC-MS system (GC, Agilent, Santa Clara, CA, USA, 7890A; MS, Agilent 5975 C).

### 4.7. Sample Sizes and Statistical Analyses

Resazurin analyses each comprised at least 12 replicates, and Student’s *t*-tests were used to compare treatments versus respective controls and between the two time points. For the RT-qPCR calculations, 4–8 biological replicates were pooled from at least 2 independent experiments to create box plots. The box plots were calculated using GraphPad Prism version 10.0.0.

## 5. Conclusions

To our knowledge, this is the first comparison of the different artemisinic drugs, ART, AS, AM, and DHA, and *A. annua* and *A. afra* tea infusions on dermal fibroblasts as they shift into myofibroblasts that precede and contribute to fibrotic tissue formation. *Artemisia annua* and *A. afra* infusions (teas), artesunate (AS), and dihydroartemisinin (DHA) significantly inhibited fibroblast viability, while artemisinin (ART) had minimal effect. AS and DHA treatments resulted in more robust effects on the transcriptional up- and downregulation of fibrotic genes compared to ART. DHA treatment resulted in both downregulation and upregulation of fibrotic genes. *A. afra* tea showed distinctly different transcriptional responses compared to *A. annua* tea. An immunofluorescence analysis indicated that AS, DHA, and *A. annua* teas notably decreased α-SMA expression, whereas ART increased α-SMA significantly. Thin-layer chromatography (TLC) analysis revealed the conversion of ART to dihydroartemisinin (dART) over time, with AS and DHA being depleted from the media within days. Artemisinin degradation was observed in fibroblast-free culture media, with dART formation even in the absence of cells. The differential effects observed among ART drugs and *Artemisia* treatments are likely regulated by different metabolites, suggesting the importance of monitoring media metabolites for understanding in vitro efficacy. These results underscore the need for more frequent drug dosing and monitoring of media metabolites to comprehensively assess ART drug efficacy.

## Figures and Tables

**Table 1:** Culture media degrades ART drugs in vitro. GC-MS analysis of culture media incubated for 4 days with 50 µM ART drugs in the absence of cells. ND = not detected.

Treatment	Day 0 (µg/mL)	Day 4 (µg/mL)
***A. annua* tea**		
ART	141.2	12.0
dART	14.5	5.1
**ART**		
ART	141.2	0.42
dART	0	0.5
**AS**		
AS	192.2	ND
dART	0	1.5
**DHA**		
DHA	142.2	ND
dART	0	6.9

**Table 2:** Primers used in the RT-qPCR analysis of drug-treated fibroblasts. NCBI Primer BLAST was used to design primers to amplify each transcript regardless of transcript variant.

Gene Name	Protein Encoded	Forward Primer (5′-3′)	Reverse Primer (5′-3′)
*ACTA2*	α-SMA	ACTGCCTTGGTGTGTGACAA	
			CACCATCACCCCCTGATGTC
*COL1A1*	Collagen 1 (α1)	GTCAGGCTGGTGTGATGGG	
			GCCTTGTTCACCTCTCTCGC
*COL3A1*	Collagen III (α1)	GGACACAGAGGCTTCGATGG	
			CTCGAGCACCGTCATTACCC
*MMP1*	Matrix metalloproteinase 1	GCATATCGATGCTGCTCTTTC	
			GATAACCTGGATCCATAGATCGTT
*MMP3*	Matrix metalloproteinase 3	ACCTGACTCGGTTCCGCCTG	
			GTCAGGGGGAGGTCCATAGAGGG
*TGFB1*	TGF-β1	CATTGGTGATGAAATCCTGGT	
			TGACACTCACCACATTGTTTTTC
*TGFB2*	TGF-β2	GAGCGACGAAGAGTACTACG	
			TTGTAACAACTGGGCAGACA
*TGFBR1*	TGF-βR1	GCAGACTTAGGACTGGCAGTAAG	
			AGAACTTCAGGGGCCATGT
*GAPDH*	GAPDH	GAGTCCACTGGCGTCTTCAC	
			TTCACACCCATGACGAACAT

## Data Availability

Data are contained within the article.
